# Crystal structure of octa­kis­(4-meth­oxy­pyridinium) bis­(4-meth­oxy­pyridine-κ*N*)tetra­kis­(thio­cyanato-κ*N*)ferrate(III) bis­[(4-meth­oxypyri­dine-κ*N*)pentakis­(thio­cyanato-κ*N*)ferrate(III)] hexa­kis­(thio­cyanato-κ*N*)ferrate(III) with iron in three different octa­hedral coordination environments

**DOI:** 10.1107/S2056989018001883

**Published:** 2018-02-02

**Authors:** Aleksej Jochim, Inke Jess, Christian Näther

**Affiliations:** a Institut für Anorganische Chemie, Christian-Albrechts-Universität Kiel, Max-Eyth Strasse 2, D-24118 Kiel, Germany

**Keywords:** crystal structure, ferrate complexes, 4-meth­oxy­pyridine, iron thio­cyanate, octa­hedral coordination

## Abstract

The crystal structure of the title salt, (C_6_H_8_NO)_8_[Fe(NCS)_4_(C_6_H_7_NO)_2_][Fe(NCS)_5_(C_6_H_7_NO)]_2_[Fe(NCS)_6_], comprises three negatively charged octa­hedral Fe^III^ complexes with different coordination environments in which the Fe^III^ atoms are coordinated by a different number of thio­cyanate anions and 4-meth­oxy­pyridine ligands. Charge balance is achieved by 4-meth­oxy­pyridinium cations. The asymmetric unit consists of three Fe^III^ cations, one of which is located on a centre of inversion, one on a twofold rotation axis and one in a general position, and ten thio­cyanate anions, two 4-meth­oxy­pyridine ligands and 4-meth­oxy­pyridinium cations (one of which is disordered over two sets of sites). Beside to Coulombic inter­actions between organic cations and the ferrate(III) anions, weak N—H⋯S hydrogen-bonding inter­actions involving the pyridinium N—H groups of the cations and the thio­cyanate S atoms of the complex anions are mainly responsible for the cohesion of the crystal structure.

## Abstract 2

The crystal structure of the title compound consists of three different negatively charged discrete octa­hedral iron(III) complexes, that are charge-balanced by 4-meth­oxy­pyridinium cations.

## Chemical context   

Recently, the synthesis of new coordination compounds based on paramagnetic metal cations has become increasingly inter­esting. In particular, compounds in which the paramagnetic metal cations are linked by small-sized anionic ligands that can mediate magnetic exchange are of special importance. For example, this can be achieved by thio- or seleno­cyanate anions that are able to coordinate to a central metal cation in different ways (Palion-Gazda *et al.*, 2015 ▸; Guillet *et al.*, 2016 ▸; Prananto *et al.*, 2017 ▸). Most of the reported compounds contain terminally N-bonded thio­cyanate ligands, whereas compounds with these ligands in a bridging mode are relatively rare. Nevertheless, the latter can be obtained by thermal decomposition of precursor complexes with terminal anionic ligands, as we have recently shown. With monodentate co-ligands, such as simple pyridine derivatives substituted in the 4-position, we were able to synthesize a number of compounds (predominantly including divalent cobalt or nickel), in which the metal cations are linked by pairs of anionic ligands into chains (Rams *et al.*, 2017**a* ▸,b* ▸; Wöhlert *et al.*, 2012 ▸; Werner *et al.*, 2015 ▸). In this context, divalent iron compounds are also of inter­est, but are scarce in comparison to divalent cobalt or nickel compounds because they are more difficult to synthesize in solution due to the poor oxidation stability of Fe^II^. Therefore, we attempted to prepare either a coordination polymer with planned composition [Fe(NCS)_2_(4-meth­oxy­pyridine)_2_]_*n*_ or a discrete complex with composition [Fe(NCS)_2_(4-meth­oxy­pyridine)_4_], which on thermal annealing might be transformed into the desired coordination polymer. 4-Meth­oxy­pyridine was selected because this ligand exhibits a strong donor substituent in the 4-position in comparison to the pyridine or 1,2-bis­(4-pyrid­yl)ethyl­ene ligands we have already investigated (Boeckmann & Näther, 2012 ▸; Wöhlert *et al.*, 2013 ▸). In the course of these investigations, we accidently obtained crystals of the title compound, (C_6_H_8_NO)_8_[Fe(NCS)_4_(C_6_H_7_NO)_2_][Fe(NCS)_5_(C_6_H_7_NO)]_2_[Fe(NCS)_6_], indicating that Fe^II^ was oxidized to Fe^III^.
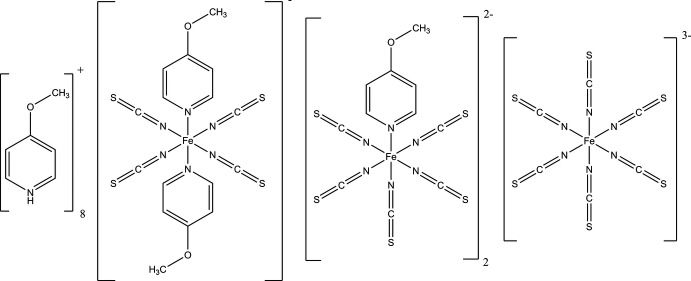


## Structural commentary   

The asymmetric unit of the title compound comprises three iron(III) cations, of which one is located on a centre of inversion (Fe3), one on a twofold rotation axis (Fe1) and one in a general position (Fe2), as well as ten thio­cyanate anions, two 4-meth­oxy­pyridine ligands and four 4-meth­oxy­pyridinium cations, one of which is disordered over two sets of sites.

**Figure 1:**
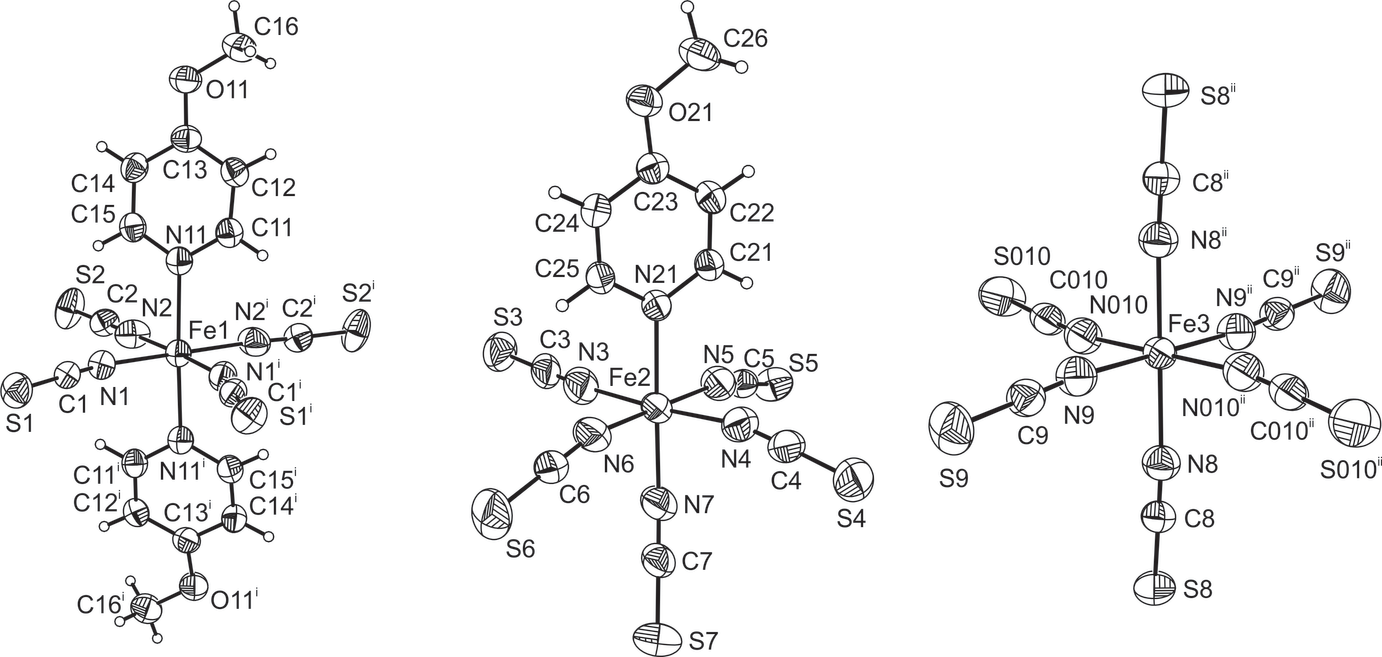
View of the three different coordination spheres of the Fe^III^ cations in the title compound. Displacement ellipsoids are drawn at the 50% probability level. [Symmetry codes: (i) 1 − *x*, *y*, 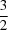 − *z*; (ii) 1 − *x*, −*y*, 1 − *z*.]

The three Fe^III^ cations form discrete anionic complexes that are charge-balanced by the 4-meth­oxy­pyridinium cations. For each of the cations, the N—H hydrogen atom was clearly located, indicating an oxidation state of +III for iron. Each of the three Fe^III^ cations shows a different octa­hedral coordin­ation environment. Fe1 is coordinated by two pairs of symmetry-related terminal-N-bonding thio­cyanate anions defining the equatorial plane of the octa­hedron, whereas the two axial positions are occupied by the N atoms of two symmetry-related 4-meth­oxy­pyridine ligands (Fig. 1 ▸). The Fe1—N distances to the anionic ligands are similar and significantly shorter than those to the neutral 4-meth­oxy­pyridine co-ligands (Table 1 ▸). Fe2 is coordinated by five crystallographically independent N-bonding thio­cyanate anions and by one 4-meth­oxy­pyridine ligand that occupies one of the axial positions (Fig. 1 ▸). The Fe2—N bond lengths are comparable to those of Fe1, except that of an equatorial thio­cyanate anion (N4) that is somewhat elongated. Inter­estingly, the distance to the N7 atom of the thio­cyanate anion that is *trans* to the 4-meth­oxy­pridine ligand is comparable to the other short Fe—N distances (Table 1 ▸). Fe3 is octa­hedrally coordinated by three pairs of N-bonding thio­cyanate anions related by a centre of inversion (Fig. 1 ▸). The Fe—N distances scatter over a wider range between 2.030 (2) and 2.075 (2) Å (Table 1 ▸). To investigate the deviations of the N—Fe—N bond angles from the ideal values, the octa­hedral angle variance σ_θ〈oct〉_^2^, which was introduced as a measure of distortion in octa­hedra (Robinson *et al.*, 1971 ▸), was calculated for each of the discrete complexes. The greatest value of σ_θ〈oct〉_^2^ is found for Fe1 (σ_θ〈oct〉_^2^ = 8.89) followed by Fe2 (σ_θ〈oct〉_^2^ = 2.34) and Fe3 (σ_θ〈oct〉_^2^ = 0.28). Thus for Fe1, the bond angles deviate more from the ideal values compared to Fe2 and Fe3, with the latter showing the smallest distortion from an ideal octa­hedron.

It is noted that a number of discrete anionic complexes based, for example, on Mn^II^ or Fe^II^ thio­cyanates, are reported in which the metal cations are four-, five-, or sixfold coordinated by anionic and additional neutral co-ligands. What makes the title compound so special is the fact that its crystal structure contains three different coordination spheres for iron in one crystal structure, suggesting a snapshot of the species that might be present in equillibrium in solution. Therefore it is not surprising that pure samples were not obtained under the given conditions. X-ray powder diffraction revealed that for all batches, large amounts of additional crystalline phases were present that could not be identified (see Fig. S1 in the Supporting information).

**Figure 2:**
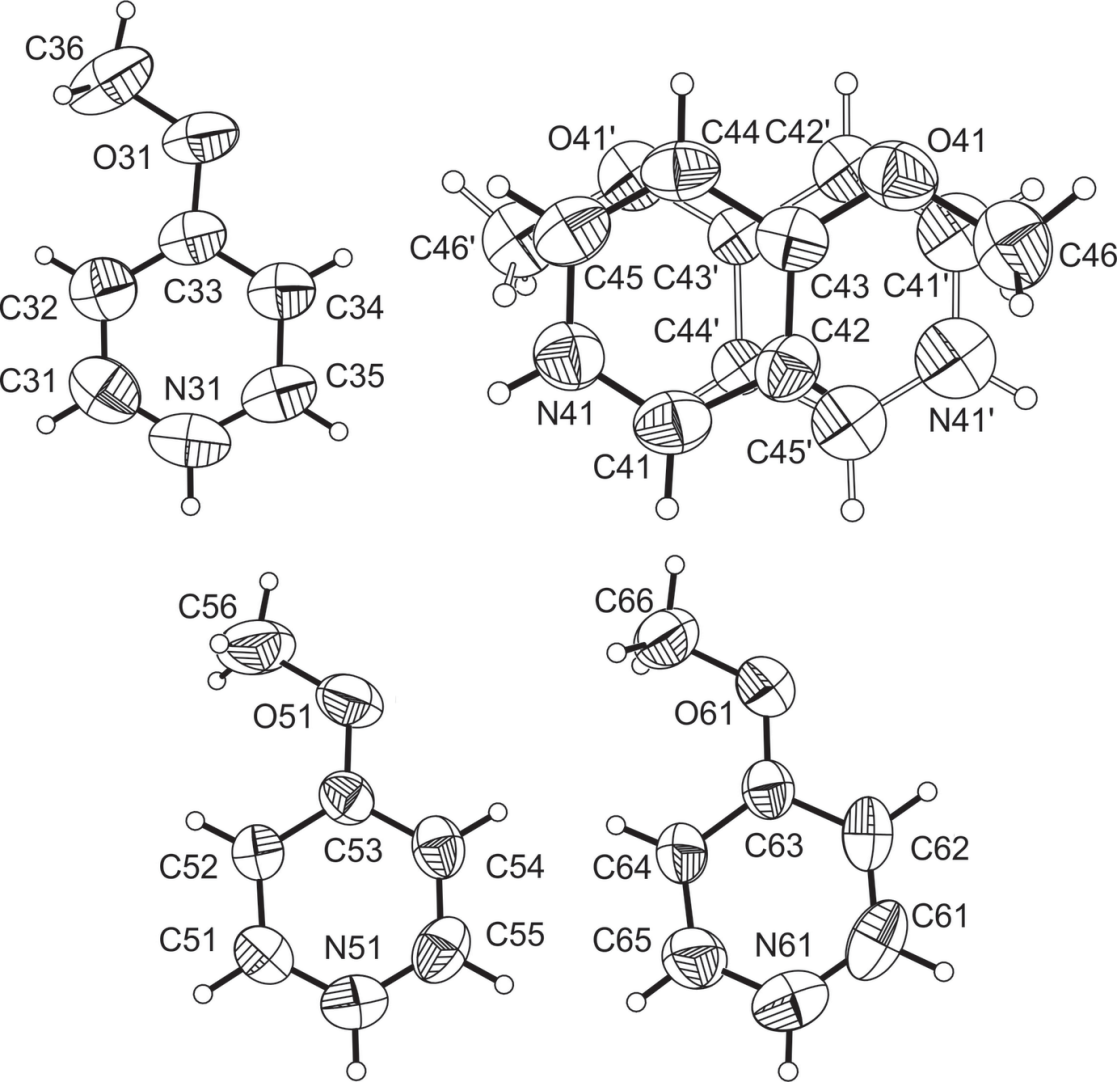
View of the four crystallographically independent 4-meth­oxy­pyridinium cations. Displacement ellipsoids are drawn at the 50% probability level. The disorder of one of the cations is shown with solid (major component) and open (minor component) bonds.

The negative charges of the anionic complexes in the title compound (–1 for Fe1, 2× −2 for Fe2 and −3 for Fe3) are compensated by eight 4-meth­oxy­pyridinium cations, of which each two are pairwise related by symmetry (Fig. 2 ▸).

## Supra­molecular features   

**Figure 3:**
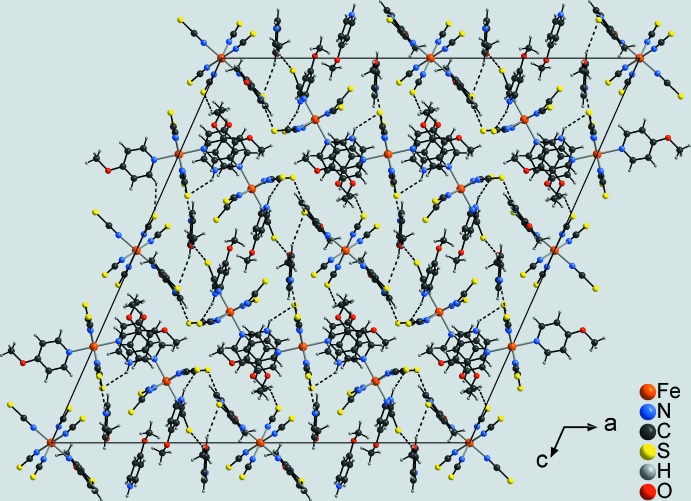
Crystal structure of the title compound in a view along [010]. Inter­molecular N—H⋯S hydrogen bonding is shown as dashed lines. The minor component of the disordered 4-meth­oxy­pyridine cation is not shown for clarity.

The discrete anionic complexes are linked with the cations through weak inter­molecular N—H⋯S hydrogen bonds between the pyridinium hydrogen atoms and the thio­cyanate sulfur atoms (Fig. 3 ▸, Table 2 ▸). The complex containing Fe3 is additionally involved in weak C_aromatic_—H⋯N hydrogen bonding. Other short contacts indicate further weak C_aromatic_—H⋯S and C_meth­yl_—H⋯S hydrogen bonds, respectively, connecting the cations and anionic complexes into a three-dimensional network.

## Database survey   

In the Cambridge Structure Database (Version 5.38, last update 2017; Groom *et al.*, 2016 ▸) only one structure containing both 4-meth­oxy­pyridine and thio­cyanate ligands is reported. It consists of discrete complexes with ruthenium(II) as the central cation coordinated by two thio­cyanate anions and four 4-meth­oxy­pyridine mol­ecules (Cadranel *et al.*, 2016 ▸). The structures of several ferrate complexes are deposited where Fe^II^ or Fe^III^ cations are present. With Fe^II^, this includes ((C_2_H_5_)_4_N)_4_[Fe(NCS)_6_] (Krautscheid & Gerber, 1999 ▸) or (2,2′-Hbpe)_4_[Fe(NCS)_6_]·4H_2_O where 2,2′-Hbpe is 1-(2-pyridin­ium)-2-(2-pyrid­yl)ethyl­ene (Briceño & Hill, 2012 ▸). Several complexes in which the Fe^III^ cation is octa­hedrally coordin­ated by six thio­cyanate anions are also known, like in (C_4_H_12_N)_3_[Fe(SCN)_6_]·4H_2_O (Addison *et al.*, 2005 ▸), or in [Ru(phen)_3_](NCS)[Fe(NCS)_4_]·H_2_O (phen: 1,10-phenanthroline), in which it is tetra­hedrally coordinated (Ghazzali *et al.*, 2008 ▸). Moreover, with pyridine as ligand and pyridinium as cation, two structures are reported with a coordination identical to those in the title compound. In the structure of (C_5_H_6_N)_2_[Fe(SCN)_5_(C_5_H_5_N)]·C_5_H_5_N, the Fe^III^ cations are octa­hedrally coordinated by five thio­cyanate anions and one pyridine ligand (Wood *et al.*, 2015 ▸). In the structure of (C_5_H_6_N)[Fe(SCN)_4_(C_5_H_5_N)_2_] the Fe^III^ cations are coordin­ated by two neutral pyridine ligands and four thio­cyanate anions (Shylin *et al.*, 2013 ▸). However, structures in which three different coordination spheres are simultaneously present like in the title compound have not been reported to date.

## Synthesis and crystallization   

Iron(II) chloride tetra­hydrate was obtained from Sigma Aldrich, potassium thio­cyanate from Fluka and 4-meth­oxy­pyridine from TCI. No further purification was carried out.

49.7 mg iron(II) chloride tetra­hydrate (0.25 mmol) and 48.6 mg potassium thio­cyanate (0.50 mmol) were reacted with 50.8 µl 4-meth­oxy­pyridine (0.50 mmol) in 2.0 ml water at room temperature. After stirring the mixture for three hours, the resulting powder was filtered off and the filtrate was let to evaporate slowly at room temperature. After several weeks single crystals suitable for single crystal X-ray analysis were obtained. The synthesis of larger and pure amounts of the title compound was not successful because in all batches additional crystalline phases were present (Supplementary Fig. S1).

## Refinement   

Crystal data, data collection and structure refinement details are summarized in Table 3 ▸. The C—H and N—H hydrogen atoms were located in a difference-Fourier map but were positioned with idealized geometry (methyl H atoms were allowed to rotate but not to tip), and refined with *U*_iso_(H) = 1.2*U*_eq_(C or N) (1.5 for methyl H atoms) using a riding model with C_aromatic_—H = 0.95 Å, C_meth­yl_—H = 0.98 Å and N—H = 0.88 Å. One of the four crystallographically independent 4-meth­oxy­pyridinium cations is disordered over two sets of sites and was refined with a split model using restraints. The sites with minor occupation (occupancy 0.22) were refined with isotropic displacement parameters, the sites of the major component with anisotropic displacement parameters.

## Supplementary Material

Crystal structure: contains datablock(s) I. DOI: 10.1107/S2056989018001883/wm5434sup1.cif

Structure factors: contains datablock(s) I. DOI: 10.1107/S2056989018001883/wm5434Isup2.hkl

Fig. S1 Experimental XRPD pattern of a representative batch obtained from the synthesis of the title compound (top) and XRPD pattern of the title compound calculated from single crystal data (bottom).. DOI: 10.1107/S2056989018001883/wm5434sup3.tif

CCDC reference: 1821019

Additional supporting information:  crystallographic information; 3D view; checkCIF report

## Figures and Tables

**Table 1:** Selected geometric parameters (Å, °)

Fe1—N2	2.030 (2)	Fe2—N5	2.045 (2)
Fe1—N1	2.038 (2)	Fe2—N4	2.074 (3)
Fe1—N11	2.1551 (19)	Fe2—N21	2.158 (2)
Fe2—N6	2.034 (3)	Fe3—N10	2.030 (2)
Fe2—N3	2.036 (3)	Fe3—N9	2.049 (2)
Fe2—N7	2.039 (3)	Fe3—N8	2.075 (2)
			
N2—Fe1—N2^i^	93.91 (15)	N6—Fe2—N4	90.10 (11)
N2—Fe1—N1^i^	176.31 (10)	N3—Fe2—N4	176.00 (10)
N2—Fe1—N1	89.62 (10)	N7—Fe2—N4	90.25 (12)
N1^i^—Fe1—N1	86.87 (12)	N5—Fe2—N4	88.73 (10)
N2—Fe1—N11^i^	87.37 (8)	N6—Fe2—N21	89.70 (9)
N2—Fe1—N11	87.05 (8)	N3—Fe2—N21	88.88 (9)
N1^i^—Fe1—N11	94.19 (8)	N7—Fe2—N21	177.30 (12)
N1—Fe1—N11	91.75 (8)	N5—Fe2—N21	90.29 (9)
N11^i^—Fe1—N11	171.82 (11)	N4—Fe2—N21	87.34 (9)
N6—Fe2—N3	91.15 (12)	N10—Fe3—N9^ii^	89.53 (9)
N6—Fe2—N7	89.08 (11)	N10—Fe3—N9	90.46 (9)
N3—Fe2—N7	93.56 (12)	N10—Fe3—N8^ii^	90.66 (9)
N6—Fe2—N5	178.84 (12)	N9—Fe3—N8^ii^	90.35 (9)
N3—Fe2—N5	90.01 (11)	N10—Fe3—N8	89.34 (9)
N7—Fe2—N5	90.87 (11)	N9—Fe3—N8	89.65 (9)

Symmetry codes: (i) 
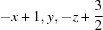
; (ii) 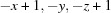.

**Table 2:** Hydrogen-bond geometry (Å, °)

*D*—H⋯*A*	*D*—H	H⋯*A*	*D*⋯*A*	*D*—H⋯*A*
C21—H21⋯N5	0.95	2.66	3.141 (3)	112
C25—H25⋯N6	0.95	2.58	3.079 (4)	113
N31—H31*A*⋯S4^iii^	0.88	2.67	3.359 (3)	136
N41—H41*A*⋯S2	0.88	2.62	3.320 (3)	137
C46—H46*C*⋯S10^iv^	0.98	2.85	3.691 (5)	144
N41′—H41*B*⋯S2^i^	0.88	2.60	3.225 (14)	129
N41′—H41*B*⋯S9	0.88	2.88	3.676 (15)	151
C42′—H42′⋯S5^v^	0.95	2.98	3.83 (3)	151
C45′—H45′⋯S1^vi^	0.95	2.86	3.370 (18)	115
C45′—H45′⋯S2^i^	0.95	2.92	3.394 (19)	112
C46′—H46*D*⋯S3	0.98	2.81	3.52 (2)	130
N51—H51*A*⋯S1	0.88	2.78	3.464 (3)	135
C54—H54⋯S8^vii^	0.95	2.97	3.885 (3)	163
C56—H56*B*⋯S7^viii^	0.98	2.90	3.793 (4)	152
N61—H61*A*⋯S8^iv^	0.88	2.62	3.419 (3)	151
C62—H62⋯S5^v^	0.95	2.93	3.831 (3)	160
C65—H65⋯N8^iv^	0.95	2.68	3.608 (4)	167

Symmetry codes: (i) 
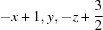
; (iii) 
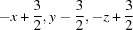
; (iv) 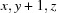; (v) 
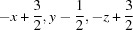
; (vi) 
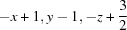
; (vii) 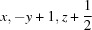; (viii) 
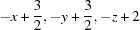
.

**Table 3:** Experimental details

Crystal data	
Chemical formula	(C_6_H_8_NO)_8_[Fe(NCS)_4_(C_6_H_7_NO)_2_][Fe(NCS)_5_(C_6_H_7_NO)]_2_[Fe(NCS)_6_]
*M*_r_	2702.57
Crystal system, space group	Monoclinic, *C*2/*c*
Temperature (K)	170
*a*, *b*, *c* (Å)	35.5034 (8), 10.5199 (1), 35.7432 (8)
β (°)	113.864 (2)
*V* (Å^3^)	12208.5 (4)
*Z*	4
Radiation type	Mo *K*α
μ (mm^−1^)	0.88
Crystal size (mm)	0.42 × 0.23 × 0.13

Data collection	
Diffractometer	Stoe IPDS2
Absorption correction	Numerical (*X-RED* and *X-SHAPE*; Stoe & Cie, 2008 ▸)
*T*_min_, *T*_max_	0.607, 0.806
No. of measured, independent and observed [*I* > 2σ(*I*)] reflections	41955, 10715, 9204
*R*_int_	0.050
(sin θ/λ)_max_ (Å^−1^)	0.595

Refinement	
*R*[*F* ^2^ > 2σ(*F* ^2^)], *wR*(*F* ^2^), *S*	0.040, 0.106, 1.04
No. of reflections	10715
No. of parameters	763
H-atom treatment	H-atom parameters constrained
Δρ_max_, Δρ_min_ (e Å^−3^)	0.86, −0.67

Computer programs: *X-AREA* (Stoe & Cie, 2008 ▸), *SHELXS97* and *XP* (Sheldrick, 2008 ▸), *SHELXL2014* (Sheldrick, 2015 ▸), *DIAMOND* (Brandenburg, 2014 ▸) and *publCIF* (Westrip, 2010 ▸).

## supplementary crystallographic information

### Crystal data

**Table:**

(C_6_H_8_NO)_8_[Fe(NCS)_4_(C_6_H_7_NO)_2_] [Fe(NCS)_5_(C_6_H_7_NO)]_2_[Fe(NCS)_6_]	*F*(000) = 5552
*M**_r_* = 2702.57	*D*_x_ = 1.470 Mg m^−^^3^
Monoclinic, *C*2/*c*	Mo *K*α radiation, λ = 0.71073 Å
*a* = 35.5034 (8) Å	Cell parameters from 41955 reflections
*b* = 10.5199 (1) Å	θ = 1.3–25.0°
*c* = 35.7432 (8) Å	µ = 0.88 mm^−^^1^
β = 113.864 (2)°	*T* = 170 K
*V* = 12208.5 (4) Å^3^	Block, brown
*Z* = 4	0.42 × 0.23 × 0.13 mm

### Data collection

**Table:**

Stoe IPDS-2 diffractometer	9204 reflections with *I* > 2σ(*I*)
ω scans	*R*_int_ = 0.050
Absorption correction: numerical (*X-RED* and *X-SHAPE*; Stoe & Cie, 2008)	θ_max_ = 25.0°, θ_min_ = 1.3°
*T*_min_ = 0.607, *T*_max_ = 0.806	*h* = −42→42
41955 measured reflections	*k* = −11→12
10715 independent reflections	*l* = −42→40

### Refinement

**Table:**

Refinement on *F*^2^	Hydrogen site location: mixed
Least-squares matrix: full	H-atom parameters constrained
*R*[*F*^2^ > 2σ(*F*^2^)] = 0.040	*w* = 1/[σ^2^(*F*_o_^2^) + (0.0524*P*)^2^ + 13.0479*P*] where *P* = (*F*_o_^2^ + 2*F*_c_^2^)/3
*wR*(*F*^2^) = 0.106	(Δ/σ)_max_ = 0.003
*S* = 1.04	Δρ_max_ = 0.86 e Å^−^^3^
10715 reflections	Δρ_min_ = −0.67 e Å^−^^3^
763 parameters	Extinction correction: SHELXL2014 (Sheldrick, 2015), Fc^*^=kFc[1+0.001xFc^2^λ^3^/sin(2θ)]^-1/4^
0 restraints	Extinction coefficient: 0.00035 (6)

### Special details

**Table:**

Geometry. All esds (except the esd in the dihedral angle between two l.s. planes) are estimated using the full covariance matrix. The cell esds are taken into account individually in the estimation of esds in distances, angles and torsion angles; correlations between esds in cell parameters are only used when they are defined by crystal symmetry. An approximate (isotropic) treatment of cell esds is used for estimating esds involving l.s. planes.

### Fractional atomic coordinates and isotropic or equivalent isotropic displacement parameters (Å^2^)

**Table:**

	*x*	*y*	*z*	*U*_iso_*/*U*_eq_	Occ. (<1)
Fe1	0.5000	0.79411 (4)	0.7500	0.03724 (12)	
Fe2	0.71066 (2)	1.07287 (4)	0.84000 (2)	0.04926 (12)	
Fe3	0.5000	0.0000	0.5000	0.03680 (12)	
N1	0.52452 (6)	0.9347 (2)	0.79210 (6)	0.0445 (5)	
C1	0.53236 (7)	1.0362 (2)	0.80667 (7)	0.0392 (5)	
S1	0.54416 (2)	1.17539 (7)	0.82712 (2)	0.05421 (18)	
N2	0.52698 (7)	0.6624 (2)	0.79435 (8)	0.0556 (6)	
C2	0.54258 (8)	0.5929 (2)	0.82173 (8)	0.0452 (6)	
S2	0.56402 (3)	0.49338 (7)	0.85800 (2)	0.0700 (2)	
N3	0.68743 (8)	0.9154 (3)	0.85615 (8)	0.0674 (7)	
C3	0.67683 (8)	0.8151 (4)	0.86277 (9)	0.0601 (8)	
S3	0.66233 (3)	0.67745 (10)	0.87176 (3)	0.0744 (3)	
N4	0.73167 (7)	1.2305 (3)	0.81932 (8)	0.0579 (6)	
C4	0.74817 (8)	1.3210 (3)	0.81490 (9)	0.0510 (6)	
S4	0.77159 (3)	1.44758 (9)	0.80971 (3)	0.0805 (3)	
N5	0.74237 (7)	0.9600 (3)	0.81615 (7)	0.0565 (6)	
C5	0.76540 (8)	0.8855 (3)	0.81352 (8)	0.0443 (6)	
S5	0.79698 (2)	0.78076 (7)	0.81075 (3)	0.0610 (2)	
N6	0.67957 (8)	1.1883 (3)	0.86335 (8)	0.0686 (7)	
C6	0.67073 (8)	1.2423 (3)	0.88703 (8)	0.0475 (6)	
S6	0.65888 (3)	1.31425 (9)	0.92017 (4)	0.0839 (3)	
N7	0.76055 (8)	1.0821 (3)	0.89460 (8)	0.0776 (9)	
C7	0.78393 (8)	1.1252 (3)	0.92523 (8)	0.0550 (7)	
S7	0.81637 (3)	1.18196 (10)	0.96765 (3)	0.0776 (3)	
N8	0.55865 (7)	0.0555 (2)	0.53986 (7)	0.0478 (5)	
C8	0.59190 (8)	0.0635 (3)	0.56455 (8)	0.0455 (6)	
S8	0.63856 (2)	0.07056 (9)	0.59923 (2)	0.0672 (2)	
N9	0.47535 (7)	0.1365 (2)	0.52431 (7)	0.0504 (5)	
C9	0.46529 (8)	0.2161 (2)	0.54081 (8)	0.0440 (6)	
S9	0.45060 (3)	0.32654 (7)	0.56329 (3)	0.0669 (2)	
N10	0.49907 (7)	−0.1274 (2)	0.54230 (7)	0.0515 (5)	
C10	0.49663 (8)	−0.2122 (3)	0.56219 (8)	0.0480 (6)	
S10	0.49314 (3)	−0.33036 (9)	0.58901 (3)	0.0790 (3)	
N11	0.44671 (6)	0.77950 (18)	0.76427 (6)	0.0391 (4)	
C11	0.40853 (7)	0.7706 (2)	0.73448 (8)	0.0420 (5)	
H11	0.4052	0.7838	0.7070	0.050*	
C12	0.37417 (8)	0.7434 (2)	0.74160 (8)	0.0439 (5)	
H12	0.3478	0.7392	0.7196	0.053*	
C13	0.37876 (8)	0.7224 (2)	0.78148 (8)	0.0435 (6)	
C14	0.41790 (8)	0.7334 (2)	0.81264 (8)	0.0448 (6)	
H14	0.4220	0.7209	0.8403	0.054*	
C15	0.45048 (8)	0.7622 (2)	0.80300 (7)	0.0419 (5)	
H15	0.4770	0.7705	0.8246	0.050*	
O11	0.34810 (6)	0.6914 (2)	0.79279 (6)	0.0558 (5)	
C16	0.30703 (8)	0.6808 (3)	0.76090 (10)	0.0643 (8)	
H16A	0.2982	0.7638	0.7479	0.096*	
H16B	0.2880	0.6524	0.7728	0.096*	
H16C	0.3071	0.6190	0.7404	0.096*	
N21	0.65815 (6)	1.07258 (19)	0.78190 (6)	0.0390 (4)	
C21	0.66158 (7)	1.0628 (2)	0.74598 (7)	0.0402 (5)	
H21	0.6884	1.0570	0.7464	0.048*	
C22	0.62854 (7)	1.0607 (2)	0.70855 (7)	0.0415 (5)	
H22	0.6325	1.0533	0.6839	0.050*	
C23	0.58915 (7)	1.0696 (2)	0.70784 (7)	0.0401 (5)	
C24	0.58506 (7)	1.0811 (2)	0.74488 (7)	0.0394 (5)	
H24	0.5586	1.0884	0.7453	0.047*	
C25	0.61953 (7)	1.0817 (2)	0.78039 (7)	0.0391 (5)	
H25	0.6163	1.0889	0.8054	0.047*	
O21	0.55360 (5)	1.06869 (18)	0.67422 (5)	0.0500 (4)	
C26	0.55563 (10)	1.0511 (3)	0.63513 (8)	0.0603 (7)	
H26C	0.5717	1.1203	0.6304	0.091*	
H26B	0.5277	1.0515	0.6135	0.091*	
H26A	0.5689	0.9696	0.6349	0.091*	
N31	0.73444 (8)	0.1116 (4)	0.61226 (9)	0.0760 (8)	
H31A	0.7380	0.1133	0.6381	0.091*	
C31	0.74033 (11)	0.0049 (4)	0.59560 (13)	0.0812 (10)	
H31	0.7470	−0.0709	0.6114	0.097*	
C32	0.73708 (10)	0.0020 (4)	0.55662 (12)	0.0735 (9)	
H32	0.7416	−0.0746	0.5450	0.088*	
C33	0.72697 (9)	0.1138 (3)	0.53394 (9)	0.0618 (8)	
C34	0.72211 (9)	0.2252 (4)	0.55238 (10)	0.0655 (8)	
H34	0.7164	0.3030	0.5376	0.079*	
C35	0.72563 (9)	0.2221 (4)	0.59177 (10)	0.0697 (9)	
H35	0.7219	0.2973	0.6046	0.084*	
O31	0.72109 (8)	0.1230 (3)	0.49454 (7)	0.0789 (7)	
C36	0.72391 (14)	0.0086 (5)	0.47393 (14)	0.1034 (15)	
H36A	0.7520	−0.0249	0.4866	0.155*	
H36B	0.7170	0.0273	0.4450	0.155*	
H36C	0.7046	−0.0546	0.4760	0.155*	
N41	0.60287 (10)	0.4088 (3)	0.79138 (10)	0.0577 (8)	0.78
H41A	0.6064	0.4147	0.8171	0.069*	0.78
C41	0.56493 (12)	0.4082 (4)	0.76217 (14)	0.0584 (9)	0.78
H41	0.5421	0.4127	0.7696	0.070*	0.78
C42	0.55780 (17)	0.4013 (4)	0.7213 (2)	0.0517 (12)	0.78
H42	0.5306	0.4029	0.7006	0.062*	0.78
C43	0.59162 (14)	0.3921 (4)	0.71152 (19)	0.0500 (9)	0.78
C44	0.63139 (17)	0.3912 (4)	0.74297 (19)	0.0556 (11)	0.78
H44	0.6548	0.3842	0.7366	0.067*	0.78
C45	0.63606 (14)	0.4005 (4)	0.7822 (2)	0.0588 (10)	0.78
H45	0.6629	0.4012	0.8035	0.071*	0.78
O41	0.58854 (16)	0.3815 (4)	0.67352 (15)	0.0650 (9)	0.78
C46	0.54772 (19)	0.3796 (5)	0.64126 (18)	0.0760 (15)	0.78
H46A	0.5316	0.3117	0.6465	0.114*	0.78
H46B	0.5496	0.3644	0.6150	0.114*	0.78
H46C	0.5343	0.4616	0.6404	0.114*	0.78
N41'	0.5283 (4)	0.3881 (14)	0.6671 (4)	0.078 (4)*	0.22
H41B	0.5037	0.3847	0.6470	0.094*	0.22
C41'	0.5639 (9)	0.387 (2)	0.6578 (8)	0.068 (6)*	0.22
H41C	0.5614	0.3769	0.6304	0.081*	0.22
C42'	0.6009 (7)	0.399 (2)	0.6891 (7)	0.056 (6)*	0.22
H42'	0.6252	0.4012	0.6840	0.067*	0.22
C43'	0.6036 (6)	0.4100 (15)	0.7292 (5)	0.040 (4)*	0.22
C44'	0.5661 (5)	0.4075 (17)	0.7368 (6)	0.038 (4)*	0.22
H44'	0.5672	0.4152	0.7637	0.046*	0.22
C45'	0.5316 (6)	0.3944 (15)	0.7055 (5)	0.068 (4)*	0.22
H45'	0.5071	0.3890	0.7101	0.081*	0.22
O41'	0.6404 (4)	0.4209 (13)	0.7590 (5)	0.060 (4)*	0.22
C46'	0.6450 (6)	0.430 (2)	0.8004 (6)	0.071 (6)*	0.22
H46D	0.6338	0.5110	0.8046	0.107*	0.22
H46E	0.6743	0.4248	0.8186	0.107*	0.22
H46F	0.6302	0.3597	0.8064	0.107*	0.22
N51	0.59908 (9)	1.0443 (3)	0.92277 (9)	0.0739 (8)	
H51A	0.5915	1.1152	0.9086	0.089*	
C51	0.59439 (14)	0.9349 (4)	0.90294 (11)	0.0872 (12)	
H51	0.5846	0.9352	0.8740	0.105*	
C52	0.60336 (12)	0.8228 (3)	0.92326 (10)	0.0726 (9)	
H52	0.5996	0.7448	0.9088	0.087*	
C53	0.61806 (8)	0.8244 (3)	0.96546 (9)	0.0534 (7)	
C54	0.62320 (9)	0.9399 (3)	0.98531 (9)	0.0586 (7)	
H54	0.6333	0.9430	1.0143	0.070*	
C55	0.61375 (9)	1.0490 (3)	0.96308 (11)	0.0658 (8)	
H55	0.6177	1.1288	0.9766	0.079*	
O51	0.62817 (7)	0.7208 (2)	0.98876 (7)	0.0753 (6)	
C56	0.62525 (15)	0.5989 (4)	0.96868 (15)	0.1087 (16)	
H56A	0.5970	0.5862	0.9484	0.163*	
H56B	0.6325	0.5308	0.9891	0.163*	
H56C	0.6442	0.5975	0.9550	0.163*	
N61	0.63662 (9)	0.7464 (3)	0.60504 (10)	0.0733 (8)	
H61A	0.6463	0.8241	0.6110	0.088*	
C61	0.65506 (10)	0.6400 (4)	0.62406 (10)	0.0738 (10)	
H61	0.6803	0.6450	0.6476	0.089*	
C62	0.63809 (9)	0.5255 (4)	0.61004 (9)	0.0637 (8)	
H62	0.6512	0.4497	0.6236	0.076*	
C63	0.60118 (9)	0.5200 (3)	0.57551 (8)	0.0516 (6)	
C64	0.58280 (9)	0.6310 (3)	0.55646 (10)	0.0603 (7)	
H64	0.5576	0.6291	0.5328	0.072*	
C65	0.60119 (10)	0.7427 (3)	0.57192 (12)	0.0746 (9)	
H65	0.5887	0.8199	0.5590	0.089*	
O61	0.58606 (7)	0.4041 (2)	0.56325 (7)	0.0689 (6)	
C66	0.54801 (13)	0.3931 (4)	0.52734 (11)	0.0837 (11)	
H66A	0.5256	0.4317	0.5328	0.125*	
H66B	0.5419	0.3032	0.5205	0.125*	
H66C	0.5507	0.4370	0.5044	0.125*	

### Atomic displacement parameters (Å^2^)

**Table:**

	*U*^11^	*U*^22^	*U*^33^	*U*^12^	*U*^13^	*U*^23^
Fe1	0.0402 (3)	0.0329 (2)	0.0371 (3)	0.000	0.0141 (2)	0.000
Fe2	0.0431 (2)	0.0689 (3)	0.0364 (2)	−0.00179 (18)	0.01665 (16)	−0.00331 (17)
Fe3	0.0354 (2)	0.0376 (3)	0.0359 (2)	0.00046 (19)	0.0128 (2)	−0.00297 (19)
N1	0.0445 (11)	0.0497 (13)	0.0382 (11)	−0.0020 (10)	0.0156 (9)	−0.0005 (10)
C1	0.0399 (12)	0.0440 (14)	0.0344 (11)	−0.0029 (10)	0.0158 (10)	−0.0017 (11)
S1	0.0670 (4)	0.0429 (4)	0.0535 (4)	−0.0062 (3)	0.0252 (3)	−0.0091 (3)
N2	0.0540 (13)	0.0515 (13)	0.0674 (15)	0.0090 (11)	0.0306 (12)	0.0143 (12)
C2	0.0533 (14)	0.0360 (13)	0.0497 (15)	0.0000 (11)	0.0243 (12)	0.0047 (12)
S2	0.1166 (7)	0.0410 (4)	0.0453 (4)	0.0038 (4)	0.0255 (4)	0.0087 (3)
N3	0.0582 (15)	0.092 (2)	0.0496 (14)	0.0032 (14)	0.0194 (12)	0.0181 (14)
C3	0.0410 (14)	0.096 (2)	0.0438 (15)	0.0074 (15)	0.0170 (12)	0.0201 (15)
S3	0.0578 (4)	0.0962 (7)	0.0681 (5)	−0.0018 (4)	0.0242 (4)	0.0257 (5)
N4	0.0519 (13)	0.0652 (16)	0.0581 (15)	−0.0100 (12)	0.0238 (12)	−0.0140 (12)
C4	0.0421 (14)	0.0595 (17)	0.0497 (15)	−0.0029 (13)	0.0167 (12)	−0.0123 (13)
S4	0.1028 (7)	0.0734 (6)	0.0786 (6)	−0.0361 (5)	0.0504 (5)	−0.0217 (5)
N5	0.0493 (13)	0.0680 (15)	0.0513 (13)	0.0066 (12)	0.0193 (11)	0.0020 (12)
C5	0.0400 (13)	0.0491 (15)	0.0397 (13)	−0.0026 (12)	0.0119 (10)	0.0029 (11)
S5	0.0506 (4)	0.0549 (4)	0.0701 (5)	0.0091 (3)	0.0168 (4)	−0.0054 (4)
N6	0.0565 (14)	0.103 (2)	0.0492 (14)	−0.0064 (14)	0.0242 (12)	−0.0212 (14)
C6	0.0417 (13)	0.0563 (16)	0.0433 (14)	−0.0018 (12)	0.0158 (11)	−0.0036 (12)
S6	0.1018 (7)	0.0733 (6)	0.1058 (7)	−0.0133 (5)	0.0722 (6)	−0.0343 (5)
N7	0.0529 (14)	0.135 (3)	0.0435 (14)	−0.0044 (16)	0.0175 (12)	−0.0062 (16)
C7	0.0465 (15)	0.076 (2)	0.0409 (15)	0.0066 (14)	0.0165 (13)	0.0043 (14)
S7	0.0787 (6)	0.0835 (6)	0.0521 (4)	−0.0013 (5)	0.0072 (4)	−0.0097 (4)
N8	0.0423 (12)	0.0512 (13)	0.0470 (12)	−0.0013 (10)	0.0150 (10)	−0.0038 (10)
C8	0.0395 (14)	0.0528 (15)	0.0443 (14)	−0.0072 (11)	0.0170 (12)	−0.0099 (12)
S8	0.0401 (4)	0.0985 (6)	0.0536 (4)	−0.0165 (4)	0.0094 (3)	−0.0112 (4)
N9	0.0472 (12)	0.0485 (13)	0.0556 (13)	0.0022 (10)	0.0209 (11)	−0.0059 (11)
C9	0.0434 (13)	0.0418 (14)	0.0479 (14)	−0.0017 (11)	0.0195 (11)	−0.0038 (11)
S9	0.0867 (6)	0.0474 (4)	0.0806 (5)	0.0039 (4)	0.0483 (5)	−0.0143 (4)
N10	0.0538 (13)	0.0515 (13)	0.0470 (12)	−0.0035 (11)	0.0181 (11)	−0.0014 (11)
C10	0.0514 (15)	0.0517 (15)	0.0363 (13)	−0.0091 (12)	0.0131 (11)	−0.0045 (12)
S10	0.0975 (7)	0.0740 (6)	0.0533 (4)	−0.0277 (5)	0.0180 (4)	0.0145 (4)
N11	0.0425 (11)	0.0344 (10)	0.0386 (10)	−0.0012 (8)	0.0145 (9)	0.0007 (8)
C11	0.0442 (13)	0.0386 (13)	0.0404 (13)	−0.0004 (10)	0.0142 (11)	0.0019 (10)
C12	0.0397 (12)	0.0447 (14)	0.0428 (13)	0.0000 (11)	0.0121 (11)	0.0015 (11)
C13	0.0412 (13)	0.0424 (13)	0.0489 (14)	0.0035 (11)	0.0205 (11)	0.0039 (11)
C14	0.0471 (13)	0.0479 (14)	0.0376 (13)	0.0048 (11)	0.0154 (11)	0.0035 (11)
C15	0.0412 (12)	0.0412 (13)	0.0399 (13)	0.0017 (10)	0.0130 (10)	0.0000 (10)
O11	0.0422 (10)	0.0753 (13)	0.0525 (11)	0.0000 (9)	0.0217 (9)	0.0084 (10)
C16	0.0409 (14)	0.087 (2)	0.0641 (19)	−0.0022 (15)	0.0210 (14)	0.0042 (17)
N21	0.0408 (10)	0.0408 (11)	0.0374 (10)	−0.0014 (8)	0.0178 (9)	−0.0010 (8)
C21	0.0406 (12)	0.0445 (13)	0.0386 (12)	0.0005 (10)	0.0191 (10)	−0.0005 (10)
C22	0.0461 (13)	0.0445 (13)	0.0378 (12)	0.0031 (11)	0.0210 (11)	0.0006 (10)
C23	0.0402 (12)	0.0381 (13)	0.0404 (13)	0.0004 (10)	0.0145 (10)	0.0016 (10)
C24	0.0392 (12)	0.0376 (12)	0.0442 (13)	0.0000 (10)	0.0199 (11)	0.0014 (10)
C25	0.0422 (12)	0.0410 (13)	0.0382 (12)	−0.0012 (10)	0.0203 (10)	0.0004 (10)
O21	0.0444 (9)	0.0641 (12)	0.0374 (9)	0.0043 (8)	0.0123 (8)	0.0020 (8)
C26	0.0614 (17)	0.076 (2)	0.0365 (14)	0.0080 (15)	0.0126 (13)	−0.0004 (14)
N31	0.0529 (15)	0.121 (3)	0.0520 (15)	−0.0178 (17)	0.0194 (12)	0.0008 (18)
C31	0.064 (2)	0.095 (3)	0.081 (3)	−0.010 (2)	0.0256 (19)	0.014 (2)
C32	0.0612 (19)	0.085 (3)	0.079 (2)	−0.0132 (17)	0.0332 (17)	−0.0039 (19)
C33	0.0499 (16)	0.082 (2)	0.0572 (17)	−0.0205 (15)	0.0251 (14)	−0.0133 (16)
C34	0.0546 (17)	0.082 (2)	0.0592 (18)	−0.0135 (16)	0.0217 (15)	−0.0070 (16)
C35	0.0484 (16)	0.101 (3)	0.0600 (19)	−0.0145 (17)	0.0217 (15)	−0.0189 (19)
O31	0.0853 (16)	0.1038 (19)	0.0560 (13)	−0.0289 (14)	0.0372 (12)	−0.0195 (13)
C36	0.101 (3)	0.131 (4)	0.097 (3)	−0.043 (3)	0.060 (3)	−0.056 (3)
N41	0.0628 (19)	0.0545 (18)	0.0594 (19)	0.0086 (15)	0.0285 (16)	−0.0037 (15)
C41	0.055 (2)	0.053 (2)	0.073 (3)	0.0061 (17)	0.032 (2)	−0.0064 (19)
C42	0.050 (3)	0.048 (2)	0.054 (3)	0.0077 (18)	0.018 (3)	−0.001 (2)
C43	0.059 (2)	0.0353 (19)	0.062 (3)	0.0048 (18)	0.030 (3)	0.006 (2)
C44	0.052 (3)	0.046 (2)	0.077 (3)	0.006 (2)	0.034 (3)	0.006 (2)
C45	0.053 (2)	0.043 (2)	0.077 (3)	0.0051 (18)	0.023 (2)	0.001 (2)
O41	0.071 (3)	0.065 (2)	0.068 (3)	0.006 (2)	0.037 (2)	0.013 (2)
C46	0.087 (4)	0.067 (3)	0.068 (3)	0.015 (3)	0.025 (3)	0.018 (3)
N51	0.086 (2)	0.0571 (16)	0.0754 (19)	0.0108 (15)	0.0294 (16)	0.0107 (14)
C51	0.124 (3)	0.077 (2)	0.0504 (18)	0.022 (2)	0.024 (2)	0.0067 (18)
C52	0.096 (3)	0.0576 (19)	0.0496 (17)	0.0106 (18)	0.0145 (17)	−0.0042 (15)
C53	0.0473 (14)	0.0591 (17)	0.0493 (15)	−0.0019 (13)	0.0147 (12)	0.0028 (13)
C54	0.0509 (15)	0.074 (2)	0.0519 (16)	0.0012 (14)	0.0215 (13)	−0.0087 (15)
C55	0.0527 (17)	0.0586 (18)	0.084 (2)	−0.0001 (14)	0.0251 (16)	−0.0165 (17)
O51	0.0763 (15)	0.0694 (15)	0.0645 (14)	−0.0028 (12)	0.0123 (11)	0.0188 (12)
C56	0.112 (3)	0.053 (2)	0.113 (3)	−0.003 (2)	−0.004 (3)	0.012 (2)
N61	0.0665 (17)	0.0770 (19)	0.085 (2)	−0.0157 (15)	0.0390 (16)	−0.0243 (17)
C61	0.0502 (17)	0.123 (3)	0.0469 (17)	−0.006 (2)	0.0188 (14)	−0.007 (2)
C62	0.0531 (16)	0.087 (2)	0.0482 (16)	0.0126 (16)	0.0180 (14)	0.0145 (16)
C63	0.0528 (15)	0.0569 (17)	0.0464 (14)	0.0053 (13)	0.0215 (13)	0.0054 (13)
C64	0.0497 (15)	0.0589 (18)	0.0610 (18)	0.0065 (14)	0.0108 (14)	0.0077 (14)
C65	0.0600 (19)	0.060 (2)	0.095 (3)	0.0046 (16)	0.0228 (19)	0.0008 (18)
O61	0.0777 (14)	0.0559 (13)	0.0672 (13)	0.0048 (11)	0.0231 (12)	0.0078 (10)
C66	0.097 (3)	0.072 (2)	0.065 (2)	−0.022 (2)	0.0148 (19)	−0.0037 (18)

### Geometric parameters (Å, º)

**Table:**

Fe1—N2	2.030 (2)	C43'—O41'	1.31 (2)
Fe1—N2^i^	2.030 (2)	C43'—C44'	1.46 (3)
Fe1—N1^i^	2.037 (2)	C44'—C45'	1.29 (2)
Fe1—N1	2.038 (2)	O41'—C46'	1.42 (2)
Fe1—N11^i^	2.1550 (19)	N51—C55	1.320 (4)
Fe1—N11	2.1551 (19)	N51—C51	1.326 (5)
Fe2—N6	2.034 (3)	C51—C52	1.353 (5)
Fe2—N3	2.036 (3)	C52—C53	1.382 (4)
Fe2—N7	2.039 (3)	C53—O51	1.329 (4)
Fe2—N5	2.045 (2)	C53—C54	1.381 (4)
Fe2—N4	2.074 (3)	C54—C55	1.358 (5)
Fe2—N21	2.158 (2)	O51—C56	1.453 (5)
Fe3—N10	2.030 (2)	N61—C65	1.334 (5)
Fe3—N10^ii^	2.030 (2)	N61—C61	1.335 (5)
Fe3—N9^ii^	2.049 (2)	C61—C62	1.349 (5)
Fe3—N9	2.049 (2)	C62—C63	1.391 (4)
Fe3—N8^ii^	2.075 (2)	C63—O61	1.332 (4)
Fe3—N8	2.075 (2)	C63—C64	1.377 (4)
N1—C1	1.171 (3)	C64—C65	1.349 (5)
C1—S1	1.614 (3)	O61—C66	1.443 (4)
N2—C2	1.166 (3)	C11—H11	0.9500
C2—S2	1.600 (3)	C12—H12	0.9500
N3—C3	1.176 (4)	C14—H14	0.9500
C3—S3	1.612 (4)	C15—H15	0.9500
N4—C4	1.162 (4)	C16—H16A	0.9800
C4—S4	1.619 (3)	C16—H16B	0.9800
N5—C5	1.163 (3)	C16—H16C	0.9800
C5—S5	1.604 (3)	C21—H21	0.9500
N6—C6	1.162 (4)	C22—H22	0.9500
C6—S6	1.599 (3)	C24—H24	0.9500
N7—C7	1.165 (4)	C25—H25	0.9500
C7—S7	1.603 (3)	C26—H26C	0.9800
N8—C8	1.156 (3)	C26—H26B	0.9800
C8—S8	1.620 (3)	C26—H26A	0.9800
N9—C9	1.161 (3)	N31—H31A	0.8800
C9—S9	1.614 (3)	C31—H31	0.9500
N10—C10	1.166 (3)	C32—H32	0.9500
C10—S10	1.605 (3)	C34—H34	0.9500
N11—C11	1.346 (3)	C35—H35	0.9500
N11—C15	1.348 (3)	C36—H36A	0.9800
C11—C12	1.373 (3)	C36—H36B	0.9800
C12—C13	1.385 (4)	C36—H36C	0.9800
C13—O11	1.346 (3)	N41—H41A	0.8800
C13—C14	1.390 (4)	C41—H41	0.9500
C14—C15	1.368 (3)	C42—H42	0.9500
O11—C16	1.447 (3)	C44—H44	0.9500
N21—C21	1.342 (3)	C45—H45	0.9500
N21—C25	1.353 (3)	C46—H46A	0.9800
C21—C22	1.377 (3)	C46—H46B	0.9800
C22—C23	1.392 (3)	C46—H46C	0.9800
C23—O21	1.345 (3)	N41'—H41B	0.8800
C23—C24	1.394 (3)	C41'—H41C	0.9500
C24—C25	1.361 (3)	C42'—H42'	0.9500
O21—C26	1.440 (3)	C44'—H44'	0.9500
N31—C31	1.326 (5)	C45'—H45'	0.9500
N31—C35	1.342 (5)	C46'—H46D	0.9800
C31—C32	1.351 (5)	C46'—H46E	0.9800
C32—C33	1.391 (5)	C46'—H46F	0.9800
C33—O31	1.341 (4)	N51—H51A	0.8800
C33—C34	1.389 (5)	C51—H51	0.9500
C34—C35	1.363 (5)	C52—H52	0.9500
O31—C36	1.435 (5)	C54—H54	0.9500
N41—C41	1.329 (5)	C55—H55	0.9500
N41—C45	1.348 (6)	C56—H56A	0.9800
C41—C42	1.381 (7)	C56—H56B	0.9800
C42—C43	1.383 (6)	C56—H56C	0.9800
C43—O41	1.323 (7)	N61—H61A	0.8800
C43—C44	1.405 (7)	C61—H61	0.9500
C44—C45	1.346 (8)	C62—H62	0.9500
O41—C46	1.442 (7)	C64—H64	0.9500
N41'—C45'	1.33 (2)	C65—H65	0.9500
N41'—C41'	1.43 (3)	C66—H66A	0.9800
C41'—C42'	1.34 (3)	C66—H66B	0.9800
C42'—C43'	1.40 (3)	C66—H66C	0.9800
			
N2—Fe1—N2^i^	93.91 (15)	C51—C52—C53	118.5 (3)
N2—Fe1—N1^i^	176.31 (10)	O51—C53—C54	116.9 (3)
N2^i^—Fe1—N1^i^	89.62 (10)	O51—C53—C52	124.1 (3)
N2—Fe1—N1	89.62 (10)	C54—C53—C52	119.0 (3)
N2^i^—Fe1—N1	176.31 (10)	C55—C54—C53	119.5 (3)
N1^i^—Fe1—N1	86.87 (12)	N51—C55—C54	120.1 (3)
N2—Fe1—N11^i^	87.37 (8)	C53—O51—C56	117.8 (3)
N2^i^—Fe1—N11^i^	87.05 (8)	C65—N61—C61	121.3 (3)
N1^i^—Fe1—N11^i^	91.76 (8)	N61—C61—C62	120.4 (3)
N1—Fe1—N11^i^	94.19 (8)	C61—C62—C63	119.0 (3)
N2—Fe1—N11	87.05 (8)	O61—C63—C64	124.5 (3)
N2^i^—Fe1—N11	87.37 (8)	O61—C63—C62	116.1 (3)
N1^i^—Fe1—N11	94.19 (8)	C64—C63—C62	119.4 (3)
N1—Fe1—N11	91.75 (8)	C65—C64—C63	118.9 (3)
N11^i^—Fe1—N11	171.82 (11)	N61—C65—C64	121.0 (3)
N6—Fe2—N3	91.15 (12)	C63—O61—C66	118.3 (3)
N6—Fe2—N7	89.08 (11)	C11—C12—H12	120.6
N3—Fe2—N7	93.56 (12)	C13—C12—H12	120.6
N6—Fe2—N5	178.84 (12)	C15—C14—H14	120.3
N3—Fe2—N5	90.01 (11)	C13—C14—H14	120.3
N7—Fe2—N5	90.87 (11)	N11—C15—H15	118.5
N6—Fe2—N4	90.10 (11)	C14—C15—H15	118.5
N3—Fe2—N4	176.00 (10)	O11—C16—H16A	109.5
N7—Fe2—N4	90.25 (12)	O11—C16—H16B	109.5
N5—Fe2—N4	88.73 (10)	H16A—C16—H16B	109.5
N6—Fe2—N21	89.70 (9)	O11—C16—H16C	109.5
N3—Fe2—N21	88.88 (9)	H16A—C16—H16C	109.5
N7—Fe2—N21	177.30 (12)	H16B—C16—H16C	109.5
N5—Fe2—N21	90.29 (9)	N21—C21—H21	118.0
N4—Fe2—N21	87.34 (9)	C22—C21—H21	118.0
N10—Fe3—N10^ii^	180.0	C21—C22—H22	120.9
N10—Fe3—N9^ii^	89.53 (9)	C23—C22—H22	120.9
N10^ii^—Fe3—N9^ii^	90.46 (9)	C25—C24—H24	120.5
N10—Fe3—N9	90.46 (9)	C23—C24—H24	120.5
N10^ii^—Fe3—N9	89.54 (9)	N21—C25—H25	118.3
N9^ii^—Fe3—N9	180.00 (12)	C24—C25—H25	118.3
N10—Fe3—N8^ii^	90.66 (9)	O21—C26—H26C	109.5
N10^ii^—Fe3—N8^ii^	89.34 (9)	O21—C26—H26B	109.5
N9^ii^—Fe3—N8^ii^	89.65 (9)	H26C—C26—H26B	109.5
N9—Fe3—N8^ii^	90.35 (9)	O21—C26—H26A	109.5
N10—Fe3—N8	89.34 (9)	H26C—C26—H26A	109.5
N10^ii^—Fe3—N8	90.66 (9)	H26B—C26—H26A	109.5
N9^ii^—Fe3—N8	90.35 (9)	C31—N31—H31A	120.5
N9—Fe3—N8	89.65 (9)	C35—N31—H31A	117.2
N8^ii^—Fe3—N8	180.0	N31—C31—H31	119.4
C1—N1—Fe1	160.8 (2)	C32—C31—H31	119.4
N1—C1—S1	178.8 (2)	C31—C32—H32	120.8
C2—N2—Fe1	175.4 (2)	C33—C32—H32	120.8
N2—C2—S2	177.7 (3)	C35—C34—H34	120.2
C3—N3—Fe2	170.6 (3)	C33—C34—H34	120.2
N3—C3—S3	179.9 (3)	N31—C35—H35	120.4
C4—N4—Fe2	168.0 (2)	C34—C35—H35	120.4
N4—C4—S4	178.9 (3)	O31—C36—H36A	109.5
C5—N5—Fe2	161.5 (2)	O31—C36—H36B	109.5
N5—C5—S5	178.6 (3)	H36A—C36—H36B	109.5
C6—N6—Fe2	160.3 (3)	O31—C36—H36C	109.5
N6—C6—S6	178.9 (3)	H36A—C36—H36C	109.5
C7—N7—Fe2	158.5 (3)	H36B—C36—H36C	109.5
N7—C7—S7	179.0 (4)	C41—N41—H41A	119.5
C8—N8—Fe3	167.4 (2)	C45—N41—H41A	119.5
N8—C8—S8	178.4 (3)	N41—C41—H41	119.2
C9—N9—Fe3	173.3 (2)	C42—C41—H41	119.2
N9—C9—S9	179.1 (3)	C41—C42—H42	121.2
C10—N10—Fe3	170.7 (2)	C43—C42—H42	121.2
N10—C10—S10	179.2 (3)	C45—C44—H44	120.2
C11—N11—C15	116.9 (2)	C43—C44—H44	120.2
C11—N11—Fe1	121.15 (16)	C44—C45—H45	119.8
C15—N11—Fe1	121.38 (16)	N41—C45—H45	119.8
N11—C11—C12	123.6 (2)	C45'—N41'—H41B	119.2
C11—C12—C13	118.7 (2)	C41'—N41'—H41B	119.2
O11—C13—C12	125.1 (2)	C42'—C41'—H41C	121.2
O11—C13—C14	116.6 (2)	N41'—C41'—H41C	121.2
C12—C13—C14	118.3 (2)	C41'—C42'—H42'	120.1
C15—C14—C13	119.3 (2)	C43'—C42'—H42'	120.1
N11—C15—C14	123.1 (2)	C45'—C44'—H44'	121.4
C13—O11—C16	117.5 (2)	C43'—C44'—H44'	121.4
C21—N21—C25	116.7 (2)	C44'—C45'—H45'	118.2
C21—N21—Fe2	122.91 (16)	N41'—C45'—H45'	118.2
C25—N21—Fe2	120.35 (15)	O41'—C46'—H46D	109.5
N21—C21—C22	124.0 (2)	O41'—C46'—H46E	109.5
C21—C22—C23	118.1 (2)	H46D—C46'—H46E	109.5
O21—C23—C22	126.1 (2)	O41'—C46'—H46F	109.5
O21—C23—C24	115.4 (2)	H46D—C46'—H46F	109.5
C22—C23—C24	118.6 (2)	H46E—C46'—H46F	109.5
C25—C24—C23	119.1 (2)	C55—N51—H51A	119.3
N21—C25—C24	123.4 (2)	C51—N51—H51A	119.1
C23—O21—C26	118.1 (2)	N51—C51—H51	119.4
C31—N31—C35	122.2 (3)	C52—C51—H51	119.4
N31—C31—C32	121.3 (4)	C51—C52—H52	120.7
C31—C32—C33	118.4 (4)	C53—C52—H52	120.7
O31—C33—C34	116.2 (3)	C55—C54—H54	120.2
O31—C33—C32	124.5 (3)	C53—C54—H54	120.2
C34—C33—C32	119.3 (3)	N51—C55—H55	119.9
C35—C34—C33	119.6 (4)	C54—C55—H55	119.9
N31—C35—C34	119.2 (3)	O51—C56—H56A	109.5
C33—O31—C36	117.7 (3)	O51—C56—H56B	109.5
C41—N41—C45	121.1 (4)	H56A—C56—H56B	109.5
N41—C41—C42	121.7 (4)	O51—C56—H56C	109.5
C41—C42—C43	117.7 (5)	H56A—C56—H56C	109.5
O41—C43—C42	123.1 (5)	H56B—C56—H56C	109.5
O41—C43—C44	117.4 (4)	C65—N61—H61A	112.2
C42—C43—C44	119.5 (6)	C61—N61—H61A	126.3
C45—C44—C43	119.6 (5)	N61—C61—H61	119.8
C44—C45—N41	120.5 (4)	C62—C61—H61	119.8
C43—O41—C46	117.5 (4)	C61—C62—H62	120.5
C45'—N41'—C41'	121.6 (18)	C63—C62—H62	120.5
C42'—C41'—N41'	118 (2)	C65—C64—H64	120.6
C41'—C42'—C43'	120 (2)	C63—C64—H64	120.6
O41'—C43'—C42'	117.9 (18)	N61—C65—H65	119.5
O41'—C43'—C44'	122.3 (15)	C64—C65—H65	119.5
C42'—C43'—C44'	119.8 (18)	O61—C66—H66A	109.5
C45'—C44'—C43'	117.3 (18)	O61—C66—H66B	109.5
C44'—C45'—N41'	123.7 (18)	H66A—C66—H66B	109.5
C43'—O41'—C46'	120.3 (16)	O61—C66—H66C	109.5
C55—N51—C51	121.6 (3)	H66A—C66—H66C	109.5
N51—C51—C52	121.2 (3)	H66B—C66—H66C	109.5

Symmetry codes: (i) −*x*+1, *y*, −*z*+3/2; (ii) −*x*+1, −*y*, −*z*+1.

### Hydrogen-bond geometry (Å, º)

**Table:**

*D*—H···*A*	*D*—H	H···*A*	*D*···*A*	*D*—H···*A*
C21—H21···N5	0.95	2.66	3.141 (3)	112
C25—H25···N6	0.95	2.58	3.079 (4)	113
N31—H31*A*···S4^iii^	0.88	2.67	3.359 (3)	136
N41—H41*A*···S2	0.88	2.62	3.320 (3)	137
C46—H46*C*···S10^iv^	0.98	2.85	3.691 (5)	144
N41′—H41*B*···S2^i^	0.88	2.60	3.225 (14)	129
N41′—H41*B*···S9	0.88	2.88	3.676 (15)	151
C42′—H42′···S5^v^	0.95	2.98	3.83 (3)	151
C45′—H45′···S1^vi^	0.95	2.86	3.370 (18)	115
C45′—H45′···S2^i^	0.95	2.92	3.394 (19)	112
C46′—H46*D*···S3	0.98	2.81	3.52 (2)	130
N51—H51*A*···S1	0.88	2.78	3.464 (3)	135
C54—H54···S8^vii^	0.95	2.97	3.885 (3)	163
C56—H56*B*···S7^viii^	0.98	2.90	3.793 (4)	152
N61—H61*A*···S8^iv^	0.88	2.62	3.419 (3)	151
C62—H62···S5^v^	0.95	2.93	3.831 (3)	160
C65—H65···N8^iv^	0.95	2.68	3.608 (4)	167

Symmetry codes: (i) −*x*+1, *y*, −*z*+3/2; (iii) −*x*+3/2, *y*−3/2, −*z*+3/2; (iv) *x*, *y*+1, *z*; (v) −*x*+3/2, *y*−1/2, −*z*+3/2; (vi) −*x*+1, *y*−1, −*z*+3/2; (vii) *x*, −*y*+1, *z*+1/2; (viii) −*x*+3/2, −*y*+3/2, −*z*+2.
